# Clot‐targeted magnetic hyperthermia permeabilizes blood clots to make them more susceptible to thrombolysis

**DOI:** 10.1111/jth.15846

**Published:** 2022-09-02

**Authors:** David Cabrera, Maneea Eizadi Sharifabad, Jacob A. Ranjbar, Neil D. Telling, Alan G. S. Harper

**Affiliations:** ^1^ School of Pharmacy and Bioengineering Guy Hilton Research Centre, Keele University Stoke‐on‐Trent UK; ^2^ School of Medicine Keele University Keele UK

**Keywords:** blood clot permeation, clot‐targeted magnetic hyperthermia, functionalized iron oxide nanoparticles, stroke, thrombolysis

## Abstract

**Background:**

Thrombolysis is a frontline treatment for stroke, which involves the application of tissue plasminogen activator (tPA) to trigger endogenous clot‐degradation pathways. However, it is only effective within 4.5 h of symptom onset because of clot contraction preventing tPA permeation into the clot. Magnetic hyperthermia (MH) mediated by tumor‐targeted magnetic nanoparticles is used to treat cancer by using local heat generation to trigger apoptosis of cancer cells.

**Objectives:**

To develop clot‐targeting magnetic nanoparticles to deliver MH to the surface of human blood clots, and to assess whether this can improve the efficacy of thrombolysis of contracted blood clots.

**Methods:**

Clot‐targeting magnetic nanoparticles were developed by functionalizing iron oxide nanoparticles with an antibody recognizing activated integrin αIIbβ3 (PAC‐1). The magnetic properties of the PAC‐1‐tagged magnetic nanoparticles were characterized and optimized to deliver clot‐targeted MH.

**Results:**

Clot‐targeted MH increases the efficacy of tPA‐mediated thrombolysis in contracted human blood clots, leading to a reduction in clot weight. MH increases the permeability of the clots to tPA, facilitating their breakdown. Scanning electron microscopy reveals that this effect is elicited through enhanced fibrin breakdown and triggering the disruption of red blood cells on the surface of the clot. Importantly, endothelial cells viability in a three‐dimensional blood vessel model is unaffected by exposure to MH.

**Conclusions:**

This study demonstrates that clot‐targeted MH can enhance the thrombolysis of contracted human blood clots and can be safely applied to enhance the timeframe in which thrombolysis is effective.

**Essentials**

- Thrombus contraction reduces the efficacy of thrombolysis by impeding Activase® permeability.
- Platelet‐targeted magnetic nanoparticles were used to deliver localized heating to blood clots.
- Clot‐targeted magnetic hyperthermia increased the permeability of contracted human blood clots.
- Magnetic hyperthermia can act as an adjuvant to thrombolysis, without damaging the endothelium.

## 1 INTRODUCTION

Ischemic strokes are a major cause of death and disability worldwide.^1^, ^2^ Clinical interventions aim to rapidly reestablish blood flow through the blocked vessel to minimize tissue damage. Current frontline treatment includes intravenous injection of the thrombolytic agent, tissue plasminogen activator (tPA), which enzymatically breaks down the fibrin scaffold holding together the blood clot to facilitate recanalization of the affected vessel. These thrombolytic drugs can be administered in out‐of‐hospital or nonspecialist clinical settings by any appropriately trained health care provider.^3^, ^4^ However, their use is currently limited by the short time window in which it is effective because clots become resistant to thrombolysis between 3 and 4.5 h after the onset of symptoms.^3^, ^4^ The reduced efficacy of thrombolysis at this stage is because of strong compaction of the blood clot, which impairs tPA diffusion into the thrombus.^3^, ^4^

Alternatively, mechanical thrombectomy can be used to surgically remove the blood clot and reopen the vessels in patients up to 24 h after symptom onset, leading to improved patient outcomes.^5^ Yet these surgical procedures require transfer to specialist hospital units, and time for patient assessment before surgery, which prolongs the delay in restoring blood flow. Therefore, optimizing current thrombolytic techniques to make them safer and more efficient could improve functional outcomes in patients.

Magnetic hyperthermia (MH) uses magnetic iron oxide nanoparticles (IONPs) to generate heat upon exposure to an alternating magnetic field (AMF). By functionalizing with peptides and antibodies that targeted cancer cells, IONPs has been used to generate a highly localized heating response that selectively trigger apoptosis and necrosis in cancer cells, while leaving the healthy tissues surrounding the tumor intact.^6^ Initial clinical trials using MH to treat glioblastoma multiform^7^ or prostate cancer have yielded promising results.^8^ Additionally, current clinical trials include the NoCanTher EU Project,^9^ that has recently commenced. More importantly, companies like MagForce AG already received a European CE Certificate to use MH hyperthermia for glioblastoma treatment.^10^, ^11^ This same company is in process of obtaining a similar license to use MH for treatment of prostate cancer in the United States.^12^ Those examples illustrate that MH is an emerging cancer therapy that will likely be used in clinical practice within the next decade.

Previous studies have demonstrated that heating platelets and red blood cells (erythrocytes) can trigger apoptosis^13^ or eryptosis, respectively.^14^ Triggering cell death in this way could clear cellular material from the compacted thrombi and thus improve the permeability of tPA into the clot, enabling the effectiveness of thrombolytic therapies to be extended over a wider timeframe. Imaging studies in patients who are experiencing an acute ischemic stroke has demonstrated that increased thrombus permeability was associated with a greater chance of rapid unblocking of the blood vessel (recanalization) after tPA treatment.^15^ Additionally, tPA activity has been shown to be enhanced by hyperthermic temperatures,^16^ suggesting that localized heating of the surface of the thrombi by IONPs could also act to optimize the enzymatic activity of tPA in the vicinity of the thrombi. Recent studies have demonstrated that it is possible to use functionalized magnetic nanoparticles that specifically bind to activated platelets as contrast agents for radiological imaging of thrombi.^17^, ^18^, ^19^, ^20^ However, no previous studies have examined the effect of MH on human blood clots.

Thrombi from ischemic stroke patients have been shown to possess a fibrinolytic‐resistant outer shell rich in densely packed platelets.^21^ Therefore, using an antibody that targets activated human platelets would provide an effective method for localizing the IONPs specifically to the offending thrombi. In this study, we fabricated IONPs labeled with the commercially available PAC‐1 antibody that specifically binds to the active form of the human platelet fibrinogen receptor, integrin αIIbβ3. Because this is only found on activated human platelets, it allows specific targeting of the IONPs to the surface of the blood clot. Here, we have tested the ability of the PAC‐1‐functionalised IONPs (f‐IONPs) to specifically deliver MH to the surface of human blood clots, and whether this could be used to enhance the thrombolytic action of tPA on contracted *ex vivo*‐generated human blood clots.

## 2 METHODS

### 2.1 Iron oxide synthesis, functionalization, and characterization

Iron oxide nanoparticles were synthesized using alkaline precipitation of iron salts through a modified version of the protocol described by Massart et al.^22^, ^23^ IONPs were coated with citrate using the protocol described by Campelj et al.^24^, ^25^ As previously described, PAC‐1 antibody was covalently attached to the citrated surface by carbodiimide activation of its carboxyl group to create the f‐IONPs.^26^ The f‐IONPs were found to be stable when dispersed into human platelet‐poor plasma (PPP) from three different donors for up to 1 h (Figure S1). Details on nanoparticle fabrication, functionalization, and characterization are provided in the Supporting Information.

### 2.2 Blood collection and generation of *ex vivo*‐derived blood clots

This study was approved by Keele University Research Ethics Committee (project reference MH‐200154, ERP2335). Blood was collected by venipuncture from healthy volunteers who had given written informed consent. Blood was mixed either with 1 part blood: 9 parts 3.8% [w v^−1^] sodium citrate solution. Platelet‐rich plasma (PRP) was isolated by centrifuging whole blood at 700 *g* for 8 min, followed by treatment with 100 μM aspirin and 0.1 U ml^−1^ apyrase. Platelet‐poor plasma was prepared by centrifuging PRP at 350 *g* for 20 min, and the supernatant was collected. In some experiments, platelets were fluorescently labeled by collecting whole blood mixed with sodium citrate containing DiOC_6_ at a final concentration of 1 μM before preparation of PRP.

Blood clots were produced by recalcifying 200 μl whole citrated blood with 20 mM CaCl_2_ in glass vials, which were then stimulated to clot by addition of 15 μM adenosine diphosphate (ADP). Formed clots were left to contract for 24 h at room temperature (RT).

### 2.3 f‐IONPs binding to activated platelets and *ex vivo*‐derived blood clots

Functionalized IONPs binding to activated platelets was assessed using a microplate‐based assay using goat anti‐mouse Alexa Fluor 488‐labeled anti‐IgM mu chain antibody (Abcam) to detect the presence of f‐IONPs. This is described fully in the supplementary materials.

Binding of f‐IONPs to *ex vivo*‐derived blood clots was assessed using alternating current (AC) susceptibility measurements and confocal imaging. AC susceptometry is a technique for measuring a magnetic property of materials called complex magnetic susceptibility (χ_AC_). Because χ_AC_ is a complex number, it is divided into a real component (χ′) related to the magnetic signal strength generated by the nanoparticles, and an imaginary component (χ″) related to the mobility of magnetic particles in a liquid media. Thus, a reduction in χ″ of magnetic nanoparticles is associated with an immobilization of magnetic nanoparticles in biological samples, such as on the surface of the clot, as described elsewhere.^27^, ^28^, ^29^

### 2.4 Thrombolysis of *ex vivo*‐derived whole blood clot

*Ex vivo*‐derived blood clots were incubated with 0.021, 0.089, or 0.33 g_Fe_ L^−1^ f‐IONPs at 37°C for 10 min and treated immediately after with 2 μg tPa only, or in combination with MH treatment using an AMF of 306 kHz and 30 mT for 30 min delivered using a NanoHeat system manufactured by NanoScience Laboratories. Control samples without further treatment or treated with 0.33 g_Fe_ L^−1^ f‐IONPs, and samples treated with 2 μg tPa and f‐IONPs, all in absence of AMF, were kept at 37°C for 30 min. The *ex vivo*‐derived blood clots were then diluted in an additional 200 μl HEPES‐buffered saline (HBS; pH 7.4, 145 mM NaCl, 10 mM HEPES, 5 mM KCl, and 1 mM MgSO_4_). A 150‐μl sample of the supernatant was extracted and diluted in a further 400 μl HBS. Absorbance of this supernatant was measured at 415 nm using a plate reader according to a previously described method.^30^ The remaining thrombi was then extracted from the glass tubes, washed with 10 ml HBS, and weighed on a microbalance.

### 2.5 Scanning electron microscopy of whole blood thrombi

Following their respective treatments, clots were fixed with White's buffer containing 2.5% [v v^−1^] glutaraldehyde for 2 h at 4°C and prepared according to OTOTO protocol^31^ (see Supporting Information). The clots were imaged using a Hitachi S4500 scanning electron microscope (SEM) operating at 5 KV. Before SEM visualization, samples were randomized by assigning a code and analyzed afterwards by an independent assessor of the team.

### 2.6 Clot permeability

Twenty microliters of DiOC_6_‐labeled PRP was recalcified with 20 mM CaCl_2_ and then stimulated with 15 μM ADP and allowed to clot for 24 h at RT. PRP clots were suspended in 200 μl PPP and incubated with 0.33 g_Fe_ L^−1^ of f‐IONPs at 37°C for 10 min. Samples were then exposed to an AMF (306 kHz, 30 mT) for 30 min using the Nanoheat system. Control samples were held at 37°C for 30 min alongside the treated samples. The samples were then extracted from the glass vials and suspended into an HBS solution containing 3 μM Rhodamine‐B‐labeled 70 kDa Dextran (Fisher Scientific UK Ltd). After 2 min of incubation at RT, the penetration of dextran into PRP clots was assessed by performing *z*‐scans using an Olympus FluoView FV 1200 confocal microscope using a slice depth of 20 μm, excitation wavelength of 473 and 543 nm, and emission wavelengths of 490 to 520 and 590 to 620 nm. Three‐dimensional (3D) images were reconstructed, and mean slice dextran fluorescence was measured using ImageJ software.

Preparation and viability assessment of 3D endothelial cell cultures human umbilical vein endothelial cells (HUVECs) were cultured on 3 mg ml^−1^ type I collagen hydrogels as previously described.^32^ The hydrogels were cultured for 3 days before being used in experiments. The 3D endothelial cell culture was incubated with 500 μl medium 200 supplemented with Low Serum Growth Supplement, and containing either no f‐IONPs or 0.089 g_Fe_ L^−1^ of f‐IONPs incubated for 10 min at 37°C. The samples were then exposed to an AMF (306 kHz, 30 mT) for 30 min or kept incubating in absence of AMF at 37°C for a similar period. The hydrogels were then stained using a live‐dead cell double staining kit II (Thermofisher Ltd). The viability of the 3D endothelial cell culture was then assessed using a FV300 confocal Olympus microscope using excitation wavelengths of 473 nm and 543 nm, and emission wavelengths of 490 to 520 nm for calcein and 590 to 620 nm for ethidium homodimer III, respectively. Cell viability was quantified in five different regions of the gel by using ImageJ software relating the proportion of live versus dead cells in each hydrogel.

### 2.7 Statistical analysis

Blood from two to five donors were used in each experiment. n signifies independently tested platelet samples. Values are reported as mean ± standard error of the mean (SErM), except for IONPs physicochemical and morphological characterization, which are mean ± standard deviation (SD). Statistical significance was assessed by one‐way anova for repeated measurements with a *post hoc* Tukey test using OriginLab Pro 8.5 software. A *p* < .05 value was considered statistically significant.

## 3 RESULTS

### 3.1 PAC1‐functionalised IONPs bind to *ex vivo*‐generated blood clots

**FIGURE 1:**
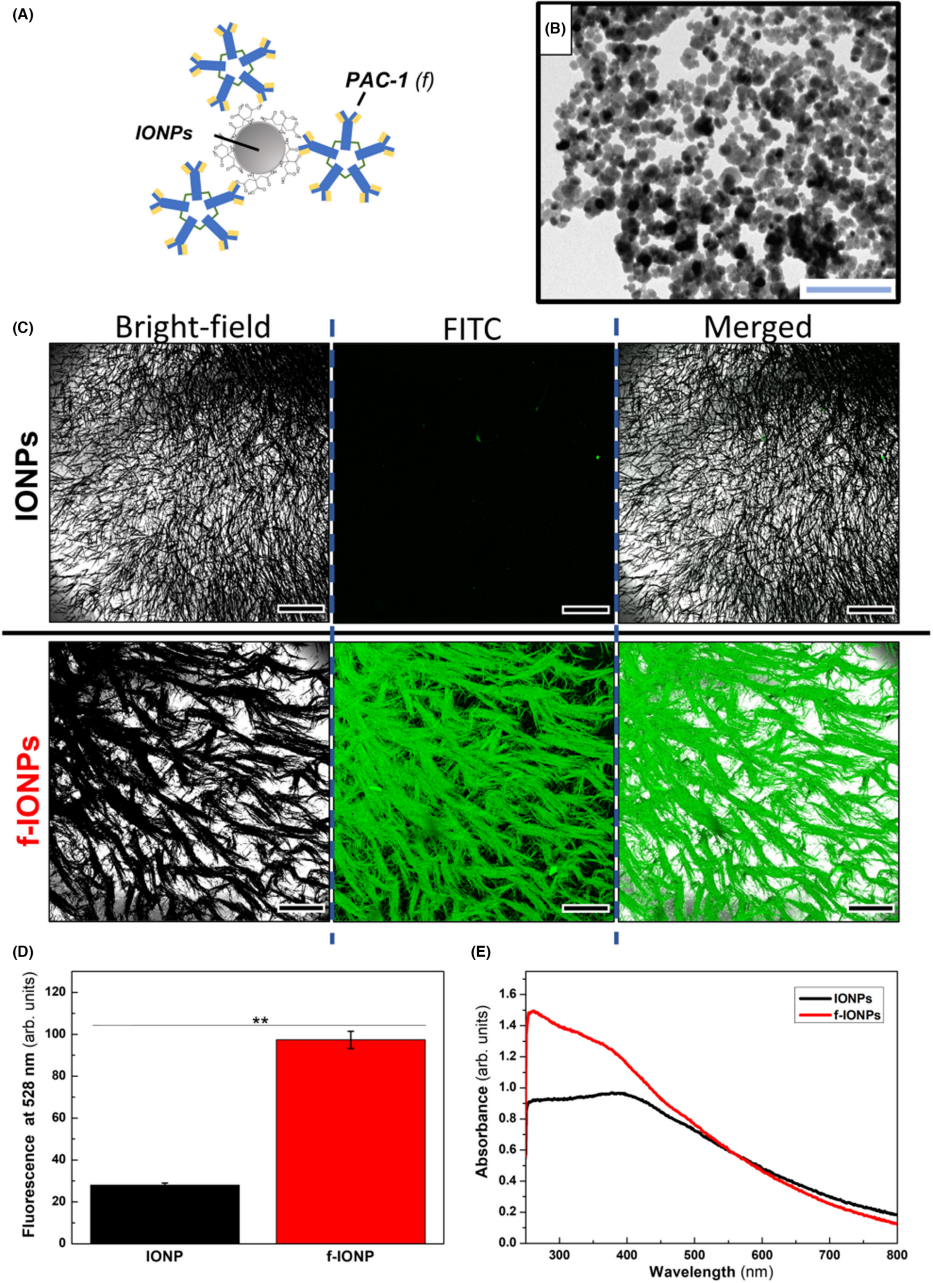
Iron oxide nanoparticles (IONPs) were successfully functionalized with PAC‐1 antibody (f‐IONPs) by covalent immobilization on nanoparticle surface. (A) Schematic representation of the functionalized iron oxide nanoparticle consisting of an iron oxide core, coating of citric acid, and conjugated with PAC‐1 antibody. (B) Transmission electron microscopy (TEM) of IONPs. (C) Confocal microscopies of IONPs and f‐IONPs after incubation with FITC‐Labeled anti‐IgM antibody. (D) Fluorescence measurements of IONPs and f‐IONPs after incubation with FITC‐labeled anti‐IgM antibody. (E) UV–vis spectroscopy of nonfunctionalized and functionalized nanoparticles. *n* = 3, **p < .01, cyan scale bar = 200 nm, black scale bar = 500 μm, error bars indicate SErM.

To develop clot‐targeted magnetic nanoparticles, citrate‐coated IONPs were functionalized with PAC‐1 antibody (illustrated schematically in Figure 1A). The production of IONPs was assessed using transmission electron microscopy (Figure 1B and Figure S1). Successful functionalization of IONPs (f‐IONPs) with PAC‐1 (an IgM antibody) was demonstrated using four different approaches. Dynamic light scattering and electrophoretic light scattering showed increased hydrodynamic size and reduced ζ‐potential after functionalization (Figure S2), consistent with antibody attachment. A FITC‐labeled anti‐IgM antibody was found to selectively label functionalized, but not untreated, nanoparticles (Figure 1C,D). Last, UV‐visible spectroscopy displayed differences in absorption traces in the UV wavelength region (Figure 1E), which is the characteristic absorption region of proteins. All these results are consistent the successful binding of the PAC‐1 antibody to the nanoparticle surface.

**FIGURE 2:**
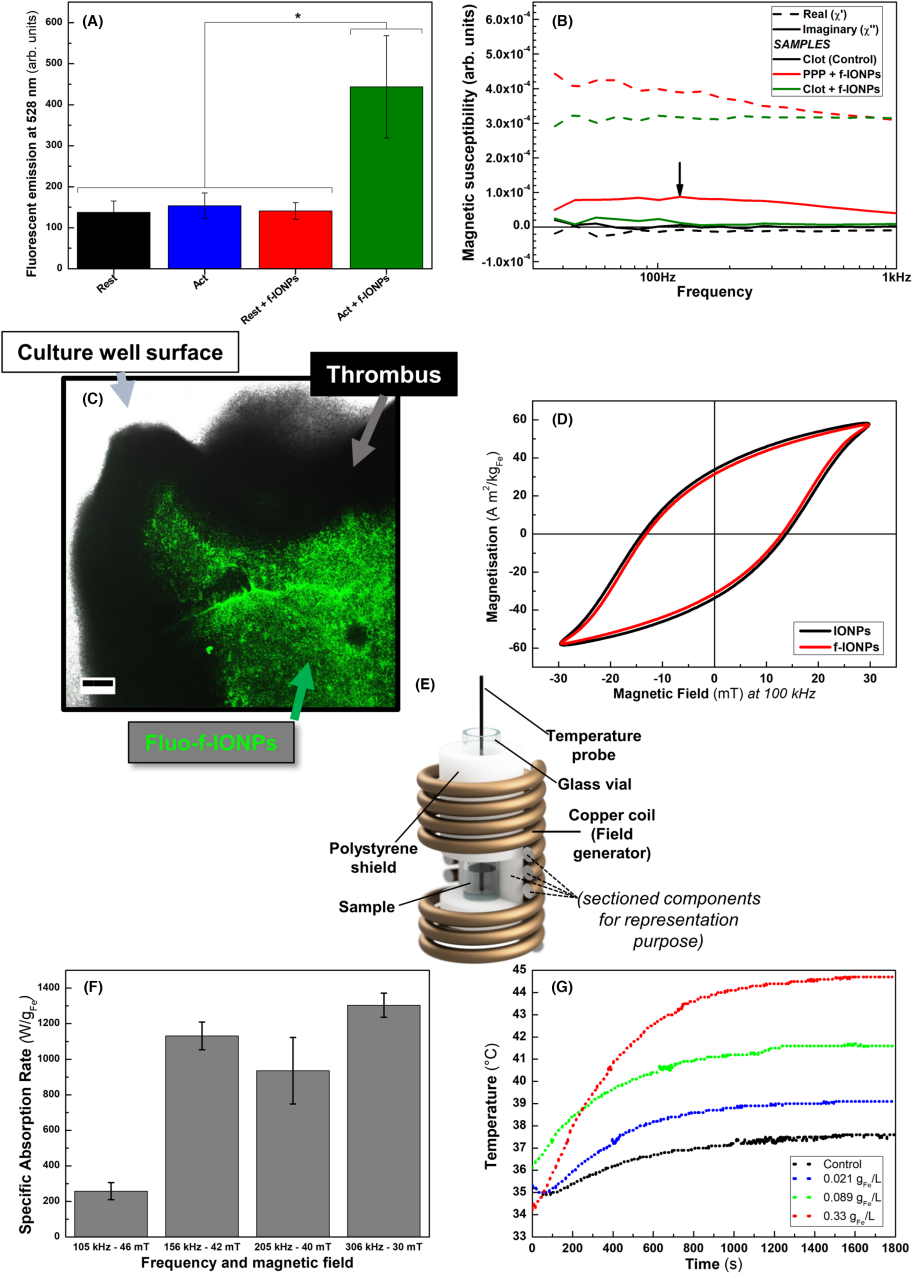
f‐IONPs labeled activated platelets and *ex vivo* blood clots and elicit hyperthermic responses upon stimulation with AMF. (A) Fluorescence measurements of resting (black and red bar) and activated (blue and green bar) incubated with f‐IONPs and, subsequently, with FITC‐labeled anti‐IgM antibody. (B) Representative AC susceptibility measurement of 200 μl PPP samples containing a human blood clot only, 0.33 g_Fe_ L^−1^ μg of f‐IONPs, or blood clots and f‐IONPs together. (C) Representative confocal image of *ex vivo*‐derived human blood clot (black shadow) preincubated with fluo‐f‐IONPs (fluo‐f‐IONPs are shown in green). (D) AC magnetization loops of 2 g_Fe_ L^−1^ of either IONPs or f‐IONPs measured at 100 kHz and 30 mT. (E) 3D representation of AMF generator system. (F) Specific absorption rate measurements of 2 g_Fe_ L^−1^ IONPs dispersed in water at 105 kHz – 46 mT, 156 kHz – 42 mT, 205 kHz – 40 mT, and 306 kHz and 30 mT. (G) Heating curves of PPP alone or containing 0.021, 0.089, and 0.33 g_Fe_ L^−1^ exposed to an AMF of 306 kHz and 30 mT for 30 min. *n* = 8 for platelet labelling measurements, *n* = 4 for confocal images with fluo‐IONPs, *n* = 3 for AC susceptometry and magnetometry measurements. *p < .05, Scale bar = 200 μm, error bars indicate SErM.

To assess whether the PAC‐1 antibody on the surface of the f‐IONPs retained its functionality, we assessed binding of f‐IONPs to resting and activated platelets using the Alexa Fluor488‐labeled anti‐IgM antibody, demonstrating an increase of fluorescence and therefore biding of nanoparticles to the well plate only when activated platelets are present (Figure 2A). To assess whether the f‐IONPs were also able to effectively label *ex vivo*‐derived human blood clots, samples were incubated with f‐IONPs and their magnetic signature detected by AC susceptibility measurements. AC magnetic susceptibility measurements enable to detect the immobilization of magnetic nanoparticles in biological entities through monitoring the imaginary part (χ″) of complex magnetic susceptibility (χ_AC_) in f‐IONPs (see Section 2). As shown in Figure 2B, F‐IONPs dispersed in PPP exhibited a prominent Brownian relaxation of the f‐IONPs, as indicated by the wide curve with a peak around 100 Hz in the imaginary part of the susceptibility (χ″, Figure 2B, black arrow). This indicates that f‐IONPs can rotate freely in PPP.^27^, ^28^, ^33^ In contrast, f‐IONPs incubated with blood clots in PPP showed a significant reduction in the imaginary part of the susceptibility signal (χ″, Figure 2B, green solid line), indicating that the f‐IONPs had become immobilized through binding to the surface of the clot. To confirm this visually, fluorescent f‐IONPs (fluo‐f‐IONPs) were prepared using a FITC‐labeled PAC‐1 antibody using the same protocol as for the untagged antibody (see Supporting Information). *Ex vivo*‐derived whole blood clots were incubated with the fluo‐f‐IONPs and these nanoparticles could be seen to strongly label the surface of the blood clot (Figure 2C). However, it was not possible to detect fluorescent signals from the thrombus core because of the known ability of hemoglobin to quench fluorescence across a wide range of wavelengths.^34^ This dense surface‐labeling of the clot with f‐IONPs would allow for localized application of MH to the surface of the blood clot to facilitate its breakdown.

To be effective at delivering MH to a blood clot, the f‐IONPs must be able to generate significant heat when stimulated with an AMF after any functionalization procedure. Because magnetic properties strongly determine f‐IONPs heating capacity, AC magnetization loops were measured before and after nanoparticle functionalization. It was found that functionalization did not significantly modify the magnetic properties of IONPs under AMF (Figure 2D). To determine the AMF conditions required for optimal heat generation, the specific absorption rate (SAR), was measured in samples contained in a polystyrene‐shielded glass vials (Figure 2E) at four different magnetic fields. SAR indicates the watts of generated thermal energy by gram of nanoparticles on exposure to an AMF. The highest SAR was found when using an AMF at 306 kHz and 30 mT (1.3 ± 0.1 kW g_Fe_^−1^; Figure 2F). These AMF parameters were then used to assess the ability of different concentrations of f‐IONPs (0.021, 0.089, and 0.33 g_Fe_ L^−1^) to heat PPP prewarmed to 37°C when exposed to this AMF for 30 min. As shown in Figure 2G, the f‐IONPs were able to increase the temperature in a dose‐dependent manner. The highest concentration of 0.33 g_Fe_ L^−1^ f‐IONPs was able to induce hyperthermic temperatures within 10 min of AMF application. Thus, this f‐IONP concentration was initially used to assess the effect of clot‐targeted MH on the clot breakdown.

### 3.2 Clot‐targeted MH enhances the thrombolytic action of tPA on contracted blood clots

**FIGURE 3:**
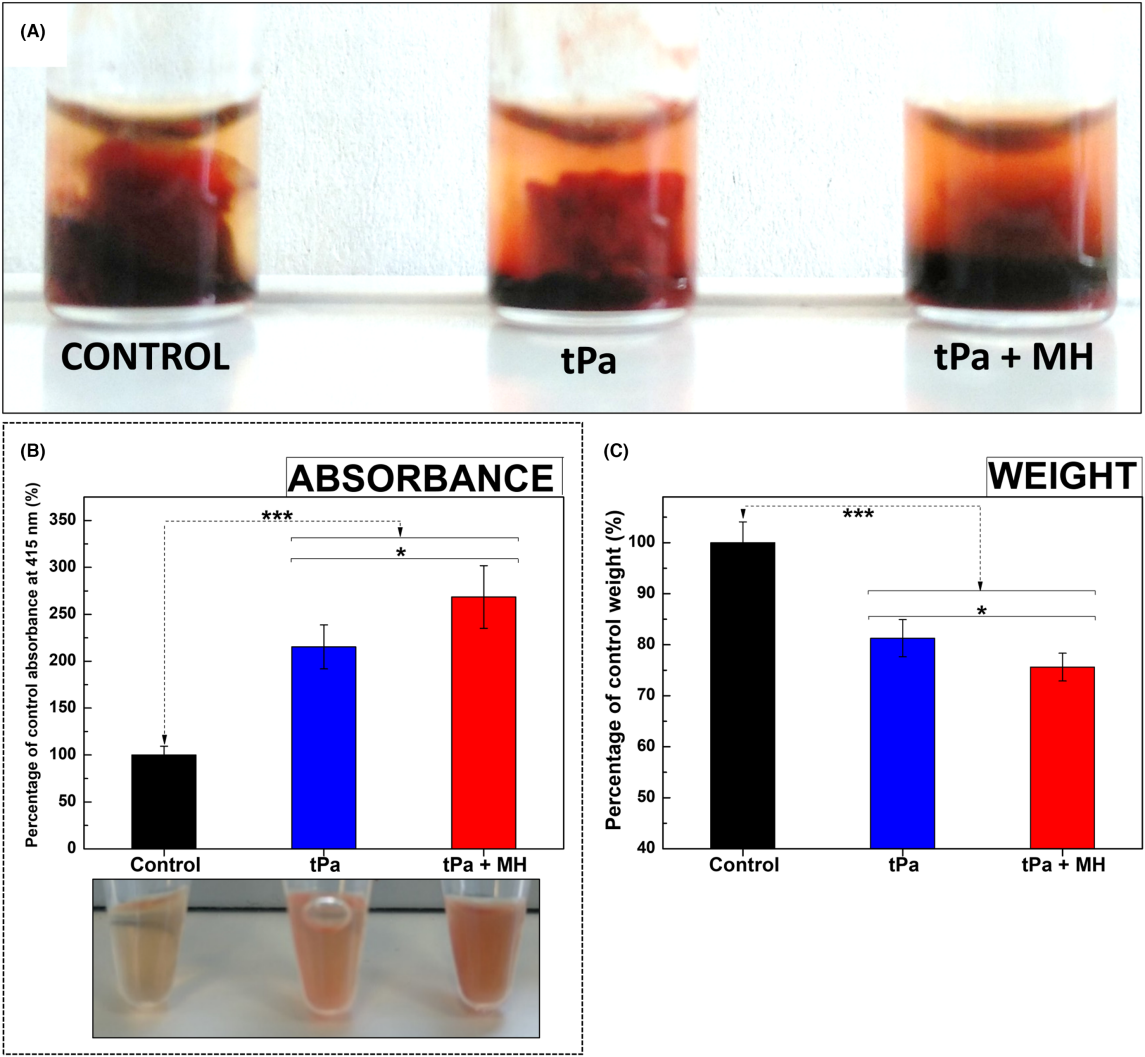
Clot‐targeted MH enhances the thrombolytic effect of highly contracted blood clots. (A) Pictures of *ex vivo*‐generated whole blood clots fabricated by 200 μl of human whole blood incubated with 0.33 g_Fe_ L^−1^ f‐IONPs without further treatment (control), treated with 2 μg of tPA (tPa), or a combination of both tPA and f‐IONPs mediated MH (tPa + MH) for 30 min. (B) Absorbance measurements (415 nm) of the supernatant of the sample after the indicated treatment. Representative images of the supernatants are also shown. (C) Mean clot weight after treatment. AMF conditions: 306 kHz and 30 mT. The clots from the control group were kept 30 min at 37°C. *n* = 15. *p < .05 between groups; ***p < .001 between groups and control; error bars indicate SErM.

To test whether clot‐targeted MH could enhance the thrombolytic activity of tPA on contracted thrombi, whole human blood was stimulated to clot by addition of ADP, and allowed to contract fully for 24 h at RT. Clots were then incubated with 0.33 g_Fe_ L^−1^ f‐IONPs alone in the absence of an AMF (control), or treated with both 0.33 g_Fe_ L^−1^ f‐IONPs and 2 μg tPA in either the presence (tPA + MH) or absence (tPA) of an AMF for 30 min. Clots treated with both MH and tPA were visibly seen to be more disrupted, with a larger sedimentation of released red blood cells observed in the plasma below the clot when compared to those treated with tPA alone (Figure 3A). These results qualitatively suggested an accelerated degradation of the blood clots when MH was used in combination with tPa. To quantitatively assess these initial visual observations, the clots were retrieved and weighed on a microbalance. Additionally, absorbance readings of plasma samples were used to assess the release of red blood cells from the clots as well as hemoglobin from damaged red blood cells using a previously described method.^30^ Plasma samples from clots treated with both tPA and MH exhibited significantly higher values of absorbance at 415 nm (275 ± 33% of control) than those treated with tPA alone (213 ± 23% of control; *n* = 15; *p* < .05; Figure 3B). These data demonstrate that there was greater release of red blood cells and hemoglobin from the clots into the surrounding plasma. This enhanced dissolution of the clots was also observed in the clot weights. MH reduced the weight of tPA + MH‐treated clots to 75.6 ± 2.7% of untreated control clots, whereas those treated with tPA alone only reduced to 81.3 ± 3.6% (*n* = 15, *p* < .05; Figure 3C). These data demonstrate that MH enhances the thrombolytic activity of tPA on contracted human blood clots.

Clot‐targeted MH treatment elicits both localized heating responses at the surface of the clot, as well as macroscopic heating of the surrounding solution. Therefore, to assess whether the enhanced thrombolysis observed was due to the macroscopic heating of the surrounding solution experiments were also performed to assess the effect of heating samples to the macroscopic temperatures elicited by MH seen in Figure 2G (up to 45°C), in the absence of f‐IONPs and an AMF. As shown in Figure S3, treatment of *ex vivo*‐generated blood clot to tPa incubated at temperatures of 42 and 45°C showed no significant differences in thrombolysis compared with the control sample held at 37°C. These results indicate that it is not the macroscopic heating of the sample by the magnetic stimulation of the f‐IONPs that elicits the enhanced thrombolytic effect of tPA observed with MH. Instead, the enhanced thrombolytic activity must be due to the localized heating responses elicited by clot‐targeted MH.

### 3.3 Thrombus‐targeted MH increases permeability to tPA

**FIGURE 4:**
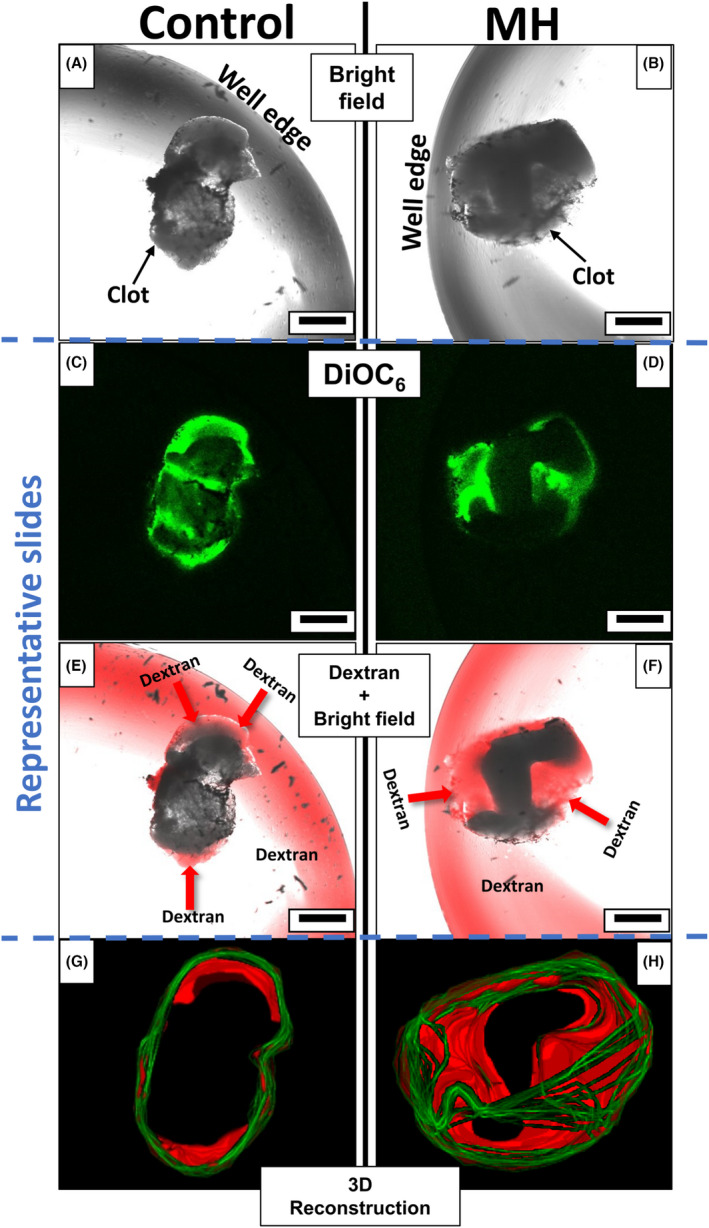
Magnetic hyperthermia increases the permeability of *ex vivo*‐derived thrombi. Thrombi were fabricated by ADP stimulation of platelet‐rich plasma (PRP). (A, C, E, G) Thrombi were treated with 0.33 g_Fe_ L^−1^ f‐IONPs in absence of AMF or (B, D, F, H) exposed to an AMF of 306 kHz and 30 mT for 30 min. The thrombi were then exposed to an HBS solution containing 3 μM rhodamine‐B‐labeled 70 kDa dextran. (A, B) Bright field images of representative thrombi. (C, D) DiOC_6_ fluorescent images of representative slides of the same thrombi. (E, F) Representative slides of combined bright field images and fluorescent dextran of clots. Red arrows indicate fluorescent dextran diffusing into the clots. (G, H) 3D reconstruction of the clot using DiOC_6_ staining to indicate the outer edge of the clot (green) and permeation of the fluorescent dextran into the thrombi (red). *n* = 10, Scale bar = 500 μm.

To examine if MH treatment was able to enhance the permeability of blood clots to tPA, *ex vivo*‐derived clots were fabricated in the same manner as before except using PRP samples instead of whole blood. PRP was used in this experiment because of the ability of hemoglobin to quench fluorescent signals,^34^ thus preventing effective fluorescent imaging in the center of whole blood clots. The thrombi were then incubated with f‐IONPs in the presence or absence of an AMF for 30 min. After this treatment, permeability of the thrombi was assessed by incubating them for 2 min with HBS containing fluorescently labeled 70 kDa dextran. Permeation of dextran into the clot was assessed using optical sectioning of the clot by a confocal microscope. This dextran is the same molecular weight as tPA, and similar to that of plasmin (83 kDa), permitting an assessment of whether thrombi‐targeted MH can enhance the permeability of the clot to tPA and plasmin. The fluorescent dextran could be seen to permeate more deeply into the clot (Figure 4). The mean slice fluorescence inside the clot was found to be significantly greater in samples exposed to MH, compared with those exposed to f‐IONPs in the absence of an AMF (147.4 ± 10.1% of control; *p* < .05, *n* = 10). In the absence of f‐IONPs and an AMF, similar experiments exposing PRP clots to macroscopic temperatures of 45°C for 30 min showed no significant increase in blood clot permeability versus control samples held at 37°C (Figure S4). These results indicate that it is the MH targeted to the surface of the blood clot that enhances thrombolysis by increasing permeability of clots to thrombolytic enzymes, and not the increase in macroscopic temperature of the solution elicited by magnetic stimulation of the f‐IONPs.

### 3.4 Clot‐targeted MH enhances tPA‐mediated fibrin breakdown and triggers erythrocyte disruption on the surface of contracted blood clots

**FIGURE 5:**
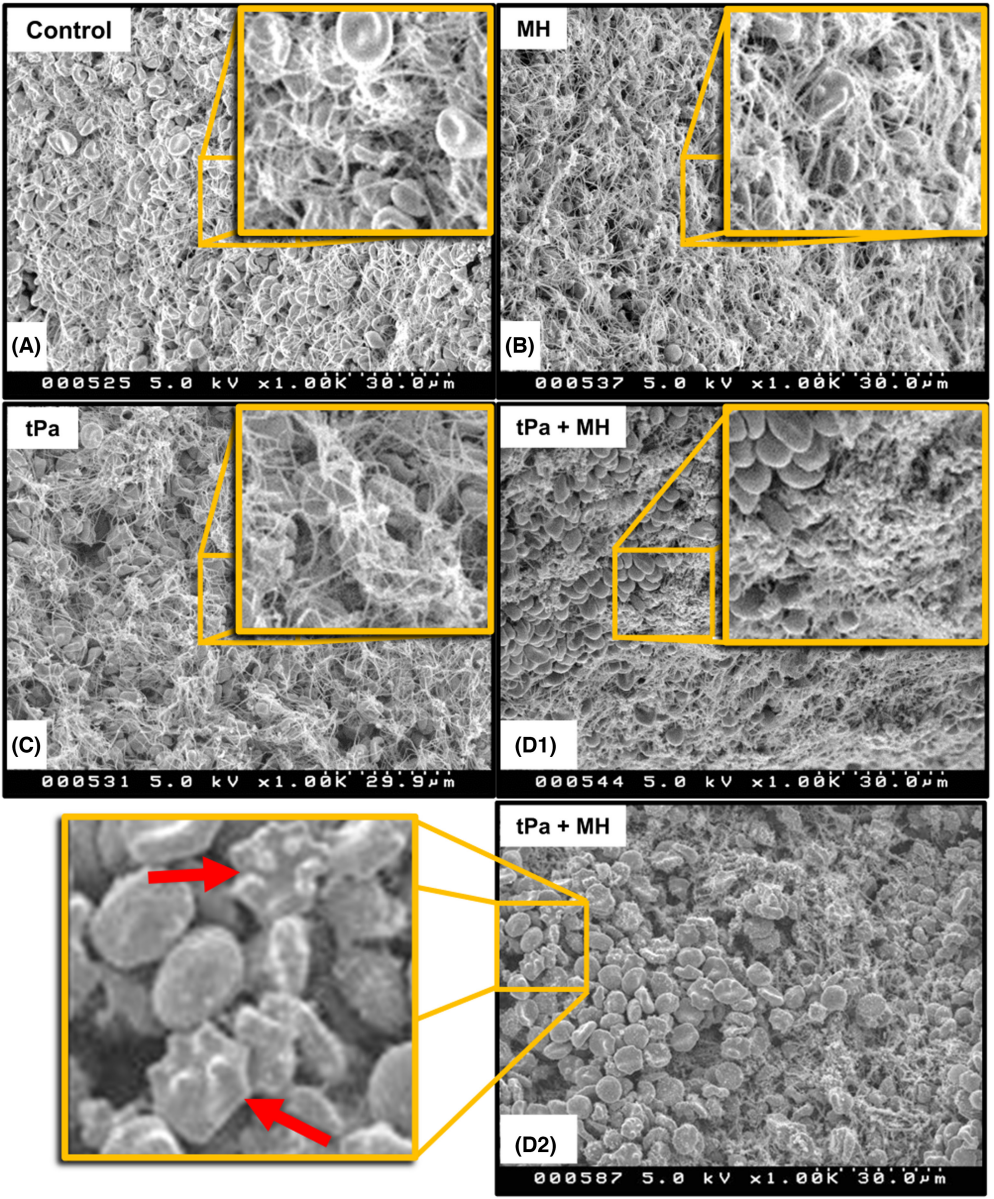
Clot‐targeted MH enhances tPA‐mediated fibrin breakdown and triggers greater red blood cell release from the surface of highly contracted *ex vivo*‐generated blood clots. Representative SEM images of *ex vivo* whole blood clots surfaces of (A) nontreated or (B) treated with 0.33 g_Fe_ L^−1^ f‐IONPs mediated MH, (C) 2 μg tPa, and (D1,2) a combined treatment with the same amount of tPa and 0.33 g_Fe_ L^−1^ f‐IONPs mediated MH for 30 min. The control group was held at a temperature of 37°C for the same period as the MH treatment. Image D1 was obtained from the same donor as figures A–C and demonstrates the presence of spherical erythrocytes and a loss of fibrin on the surface of these clots. Image D2 is taken from another donor and shows the presence of erythrocytes with membrane blebbing (red arrows). AMF conditions: 306 kHz and 30 mT. *n* = 3.

Scanning electron microscopy was used to assess how MH was assisting tPA in triggering breakdown of the contracted blood clots. Contracted blood clots were treated with either tPA alone, MH alone or both in combination before fixation and imaging by SEM. An untreated clot was also used a control. As expected, treatment of tPA alone caused a loss of fibrin on the surface of the blood clot when compared with the untreated control (Figure 5A,C). Clots exposed to MH in the absence of tPA were observed to have less red blood cells present on the surface^14^ (Figure 5B). However, fibrin fibers appeared to be unaffected by MH alone. Last, combined treatment of tPA and MH appeared to remove almost all of the visible fibrin fibers from the surface suggesting that MH can enhance the thrombolytic activity of tPA (Figure 5D1). Additionally, as with MH treatment alone, red blood cells were seen to be smaller, more spherical structures (Figure 5D1) with a few cells showing membrane blebbing in some samples, potentially indicative of eryptosis^9^ (red arrows in Figure 5D2). These results are consistent with previous studies that have shown that heating enhances the thrombolytic actions of tPA^16^ and triggers eryptosis.^14^ However, we cannot rule out the possibility that the change in erythrocyte morphology observed is due to heat‐induced necrotic damage of these cells. These data also indicate that the localized heating of the clot does not adversely impact fibrin structure or cause it denaturation to reduce its susceptibility to fibrinolysis. The results presented here suggest that a local heating effect induced by MH at the surface of the clot could elicit this adjuvant effect on thrombolysis both by directly increasing the fibrinolytic activity of tPA,^11^ as well as enhancing tPA diffusion into the clot through disruption of erythrocytes on the clot surface.

### 3.5 MH using an optimized dose of f‐IONPs has no significant effect on the viability of the surrounding endothelium

**FIGURE 6:**
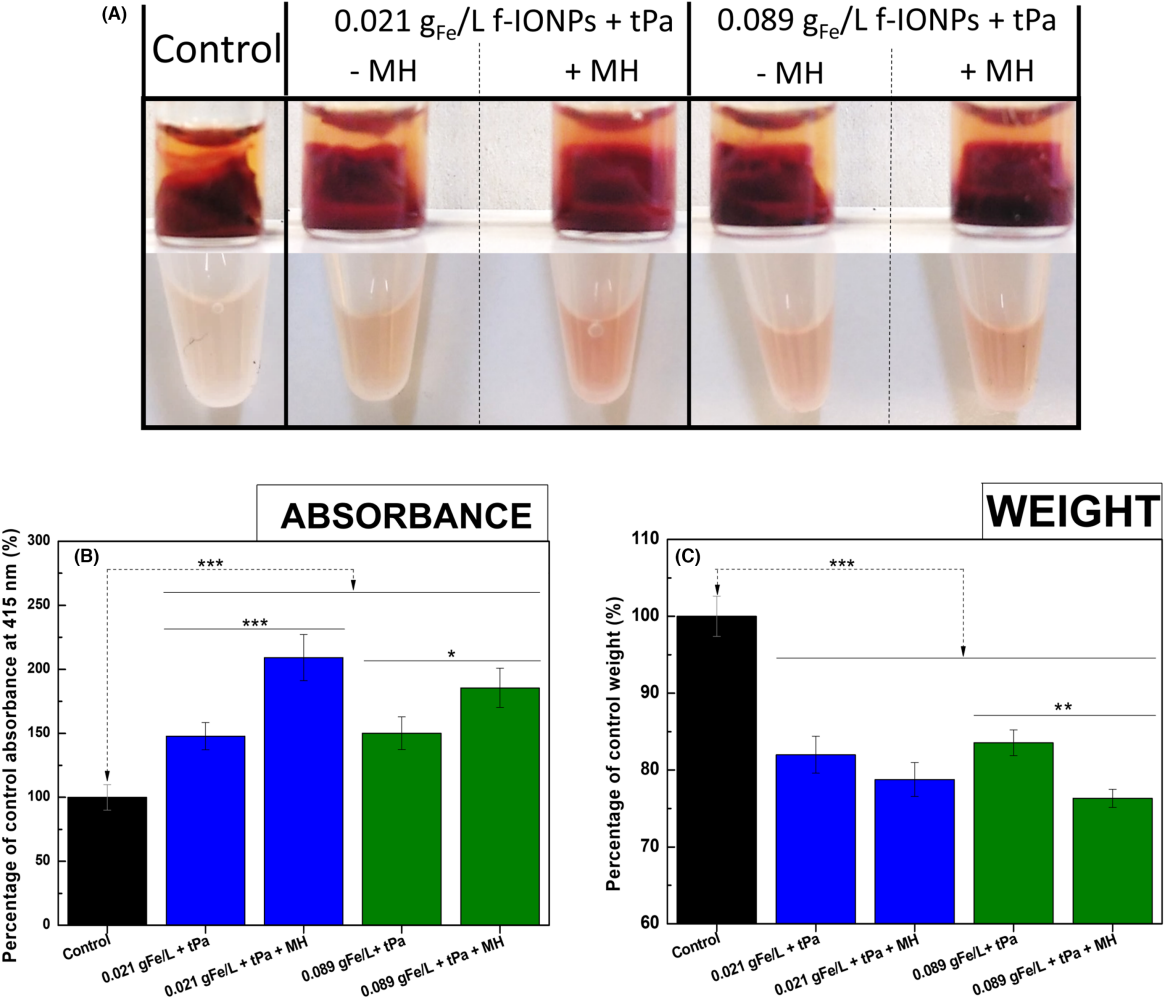
MH using an optimized dose of f‐IONPs continues enhancing thrombolytic effect of highly contracted blood clots. (A) Pictures of *ex vivo*‐generated whole blood clots fabricated by 200 μl of human whole blood with no treatment (control), incubated with 0.021 g_Fe_ L^−1^ f‐IONPs or 0.089 g_Fe_ L^−1^ f‐IONPs, both in combination with 2 μg of tPA (tPa) only (‐MH) or with AMF exposure for 30 min (+MH). Representative images of the supernatants are also shown. (B) Absorbance measurements (415 nm) of the supernatant of the sample after the indicated treatment. (C) Mean clot weight after treatment. *n* = 11 *p < .05, **p < .05, ***p < .001; error bars indicate SErM.

**FIGURE 7:**
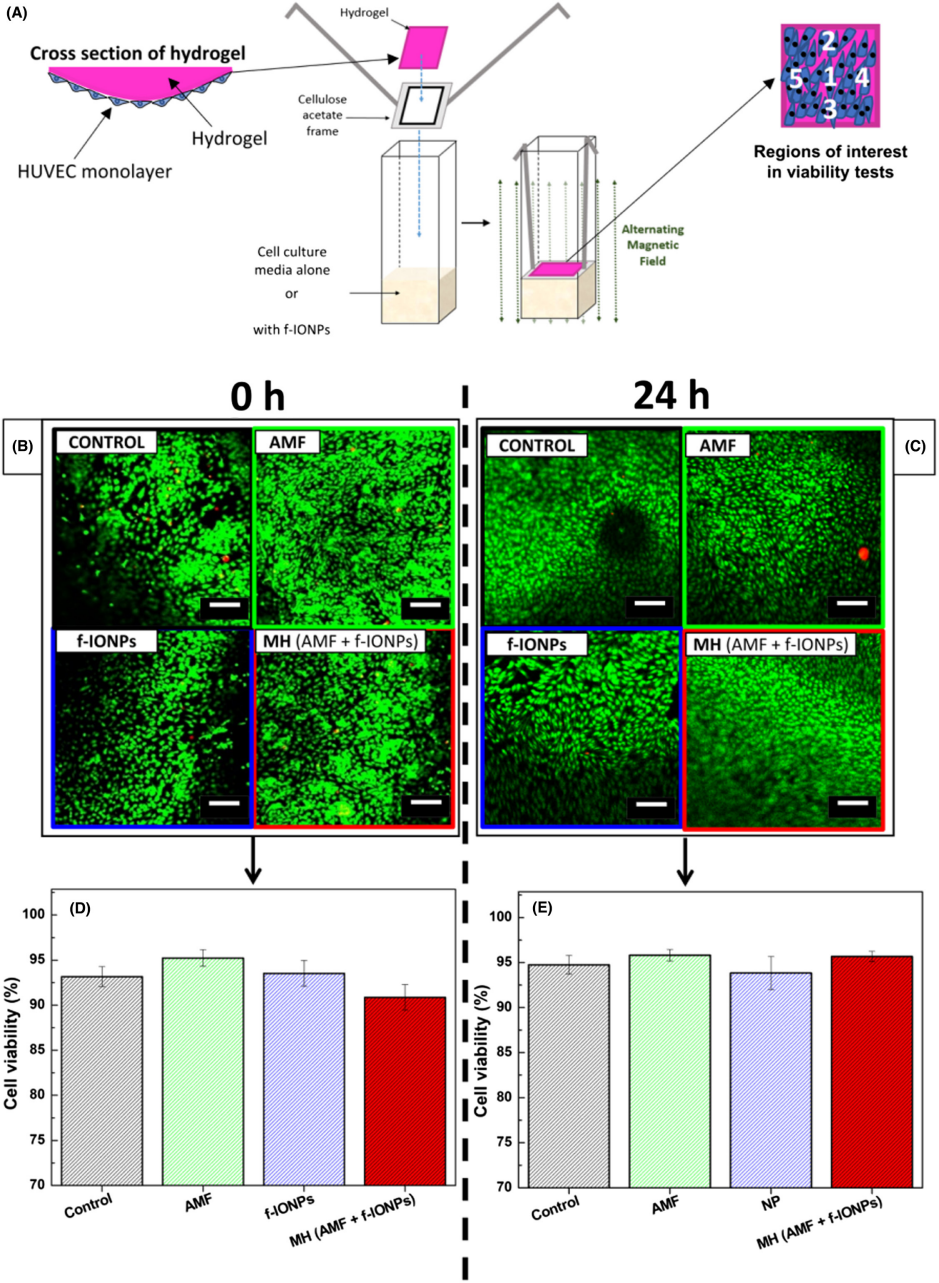
MH using an optimized dose of f‐IONPs does not affect the viability of the surrounding endothelium. (A) Schematic representation of the experimental setup to assess HUVEC viability after exposure to magnetic hyperthermia. A 3D human tissue engineered intimal layer (TEIL) model was produced by growing a HUVEC monolayer on the surface of a 3D collagen hydrogel. The TEIL was then exposed to cell culture media in the presence or absence of either f‐IONPs and AMF.The hydrogels were then subsequently stained with a fluorescent live/dead cell stain kit. Viability of HUVECs was imaged and averaged over five different regions of interest on the surface of each TEIL sample as shown. (B, C) Representative images of the live/dead cell staining observed in TEIL treated with either nothing additional (control), an AMF alone (AMF), 0.089 g_Fe_ L^−1^ f‐IONPs in the absence of an AMF (f‐IONPs), and 0.089 g_Fe_ L^−1^ f‐IONPs in the presence on AMF to elicit MH either immediately after the experiments or (B) 24 h after (C). (D, E) Mean HUVEC viability of the TEIL immediately after the experiments (D) or 24 h after the treatment (E). AMF conditions: 306 kHz and 30 mT. *n* = 8, Scale bar = 200 μm, error bars indicate SErM.

Because MH could elicit unintended thermal damage to the surrounding blood vessel, we next sought to identify the minimal MH response required to enhance thrombolysis. By decreasing the f‐IONP dose used to 0.021 and 0.089 g_Fe_ L^−1^, we previously demonstrated this reduced the macroscopic heating of a PPP sample during a 30‐min MH treatment to below 39°C and 42°C respectively (Figure 2G). As shown in Figure 6, MH induced by these lower f‐IONPs concentrations was still able to enhance the thrombolytic action of tPA, with both doses significantly increasing the release of erythrocytes into the PPP on treatment (209.1 ± 18.1% and 185.5 ± 15.4% of control, respectively; *n* = 11; Figure 6B). Although the MH elicited by both f‐IONP doses also tended to enhance the tPA‐mediated reduction in clot weight, this was only statistically significant for the higher dose of 0.089 g_Fe_ L^−1^ (Figure 6C). These data suggest that this lower dose can still enhance the thrombolytic action of tPA while maintaining the temperature of the surrounding plasma below 42°C. This is lower than the 45°C elicited in our earlier experiments, as well as previous studies using untargeted magnetic nanocubes functionalized with tPA to enhance dissolution of murine blood clots (49°C).^35^ These data therefore demonstrate that surface‐targeted MH can elicit the same enhancement of thrombolysis, while limiting macroscopic rises in the temperature of the surrounding plasma. Additionally, experiments demonstrated that exposure of whole human blood samples to this concentration IONPs in presence or absence of the AMF did not alter the coagulation time of recalcified whole human blood samples, indicating that the IONP core or MH of blood did not induce coagulation on its own (Figure S5). To assess if this heating response could damage the surrounding blood vessel, experiments were performed to assess whether the heating of PPP samples by f‐IONP could impact upon the viability of a 3D HUVEC culture. The HUVEC‐coated surface was exposed to a 500‐μl cell culture sample containing either no f‐IONPs or 0.089 g_Fe_ L^−1^ f‐IONPs, and incubated for 30 min in either the presence or absence of the same MH treatment previously used (Figure 7A). HUVEC cell viability was then assessed using live/dead cell staining to assess whether MH could damage the endothelial lining (Figure 7B). As shown in Figure 7D, HUVEC hydrogels treated with MH mediated by 0.089 g_Fe_ L^−1^ f‐IONPs showed no significant difference in cell viability (90.9 ± 1.4%, *n* = 8) in comparison with HUVECs exposed to media containing no f‐IONPs and not subjected to an AMF (93.2 ± 1.1%, *n* = 8). Additionally, exposure to either the AMF or f‐IONPs alone did not produce a significant reduction in cell viability (95.2 ± 0.9% and 93.5 ± 1.4% cell viability, respectively; *n* = 8). Additionally, similar tests performed 24 h after each treatment lead to insignificant differences between the control (94.7 ± 1% of cell viability; *n* = 8; Figure 7E) and samples treated with AMF, f‐IONPs, or the combination of AMF and f‐IONPs (95.8 ± 0.6; 93.8 ± 1.8 and 95.7 ± 0.6 of cell viability, respectively; *n* = 8; Figure 7E). These data therefore indicate that the macroscopic heating of PPP could enhance thrombolysis without impacting on the viability of the nearby endothelium.

## 4 DISCUSSION AND CONCLUSIONS

The time window to successfully treat ischemic strokes using thrombolytic therapies is limited to 3 to 4.5 h. After this period, the blood clot becomes resistant to thrombolysis from the inability of tPA to penetrate the highly restricted intercellular spaces in the contracted clot. Both the platelets as well as the presence of polyhedrocytes (erythrocytes deformed by clot contraction) have been suggested to impair thrombolysis by preventing tPA penetration into the core of the clot.^4^ Specific heating of the clot surface increased clot permeability both by directly increasing the fibrinolytic activity of tPA, as well as through disrupting erythrocytes on the surface to increase clot permeability to tPA. Interestingly, increased thrombus permeability was previously found to be predictive of successful early recanalization of obstructed blood vessels.^15^ Although the targeted heat delivery elicited by the f‐IONPs to the surface of the clot could disrupt its structure, the macroscopic heating of the surrounding plasma was crucially not sufficient to affect the viability of endothelial cells, indicating that this thrombus‐targeted MH should not elicit adverse side effects through damaging the surrounding vasculature. The feasibility of using MH to induce localized heating in the brain for therapeutic applications has been previously demonstrated in humans.^36^ In this case, a magnetic field generator was safely used to apply MH as an anticancer therapy that increased survival times in glioblastoma patients. The results reported here are the first to demonstrate the potential of clot‐targeted MH to enhance thrombolysis in humans and suggests that this could be used as a viable method to improve outcomes in stroke patients. Because the PAC‐1 antibody is specific for human platelets, it is not possible to currently conduct experiments on *in vivo* thrombosis models. Additional work will be required to produce, optimize the magnetic properties, and validate the effectiveness of analogous JON/A‐functionalized IONPs to elicit clot‐targeted MH before can be conducted to confirm the *in vivo* efficacy and safety of this treatment.^37^ The ability of MH to enhance the susceptibility of contracted blood clots to tPA, could provide us with a method to increase the time window in which clot lysis is effective. This is especially important for communities who do not have access to the specialist personnel or surgical facilities required to undertake thrombectomy. Thus, clot‐targeted MH could significantly reduce the incidence of death and disability in people experiencing ischemic stroke globally.

Further development of the PAC‐1‐functionalized IONPs could provide additional benefits of this magnetic nanotechnology in the diagnosis and treatment of ischemic stroke. This includes using the nanoparticles to specifically target release of tPA at the clot, thus reducing the risk of intracranial hemorrhage from systemic application of this drug.^35^ Additionally, further development of the composition and size of the magnetic nanoparticle core could allow these clot‐targeted nanoparticles to be used as a contrast agent to facilitate delivery of the magnetic nanoparticles into occluded blood vessels^38^ the imaging of blood clots,^39^ or for magnetic filtration of labeled emboli to prevent these lodging in other brain regions or in the lungs, minimizing the secondary consequences of thrombolysis.^40^ Last, we recently demonstrated citrate‐coated magnetic nanoparticles such as these are able to slow platelet activation, which could further prevent additional thrombi forming.^41^ Development of these multifunctional magnetic nanoparticles could provide a theranostic agent that is able to rapidly target, treat and facilitate the removal of thrombi from the cerebral circulation.

## AUTHOR CONTRIBUTIONS

A. G. S. Harper and D. Cabrera secured funding for this research. A. G. S. Harper, D. Cabrera, and N. D. Telling conceived this research. M. Eizadi Sharifabad fabricated the iron oxide nanoparticles cores. D. Cabrera functionalized and characterized the iron oxide nanoparticles. D. Cabrera, A. G. S. Harper, and J. A. Ranjbar performed the experiments. A. G. S. Harper and D. Cabrera wrote the paper, which was amended and accepted by all authors.

## CONFLICT OF INTEREST

The authors have no conflict of interest to disclose.

## Supporting information


Appendix S1
