# Supplementary material for: Clot‐targeted magnetic hyperthermia permeabilizes blood clots to make them more susceptible to thrombolysis
Source: J Thromb Haemost. 2022 Sep 2;20(11):2556–70. doi: 10.1111/jth.15846 (PMC9826519; doi:10.1111/jth.15846)
Supplement: Supplementary file 1 — Appendix S1 [file JTH-20-2556-s001.pdf]

Supporting Information

Clot-Targeted Magnetic Hyperthermia Permeabilizes Blood Clots to Make Them More Susceptible to Thrombolysis

*David Cabrera, Maneea E. Sharifabad, Jacob A. Ranjbar, Neil D. Telling, Alan G. S. Harper**

Dr. D. Cabrera, Dr. M. E. Sharifabad, Jacob A. Ranjbar, Prof. N. D. Telling
School of Pharmacy and Bioengineering, Keele University, Guy Hilton Research Centre, Thornburrow Drive, Hartshill, Stoke-on-Trent ST4 7QB United Kingdom

Dr. A. G. S. Harper
School of Medicine, Keele University, Staffordshire, ST5 5BG, United Kingdom
E-mail: a.g.s.harper@keele.ac.uk

IONPs synthesis

Iron (III) chloride hexahydrate (FeCl3. 6H2O, 22.95 g, 0.08 mol, Sigma) and iron (II) chloride tetrahydrate ($FeCl_2$. 4H2O, 8.46 g, 0.04 mol, Sigma) was dissolved in 500 mL of degassed deionised water under nitrogen environment. Nitrogen was used in preparations to prevent oxidation of the iron species in aqueous environments. In the presence of dissolved oxygen molecules, the resulting magnetic colloids are usually reddish-brown indicating contamination of the colloids with other iron oxides (γ-fe2O3) as a result of strong oxidation of magnetite. The brown solution was heated to 80°C whilst stirring under nitrogen. Aqueous ammonium hydroxide (50 mL, 25% w v^{-1} NH_4OH) was added drop wise to the mixture over a 30 minuets period. The reaction was allowed to proceed for further one hour and then, the reaction mixture was transferred to a conical flask and allowed to rest on a magnet. The black product was washed to neutral pH with distilled, deionised water via magnetic separation. The final product has pH~7. The obtained magnetic fluid was washed with ddH_2O until debris was not observed under transmission electron microscopy (TEM- JEOL JEM-1230). IONPs core size was determined using TEM operated at 100 KV measuring the diameter of 200 nanoparticles using

ImageJ software, obtaining a value of 21 ± 7 nm (Fig. S1). The iron concentration of IONPs was quantified using the Ferrozine method[1]. Briefly, IONPs were digested in 6 M nitric acid at 60°C overnight. Afterwards, digested samples were incubated in 3.3 M hydroxylamine hydrochloride at RT for 2.5 hours to reduce Fe^{3+} cations to Fe^{2+}. Finally, samples were incubated in the dark in a solution containing 1.75 mM ferrozin and 0.1 mM HEPES for 1 hour at RT, and their absorbance were measured in a plate reader at 562 nm. The absorbance was then compared with a calibration curve obtained using a standard iron solution.

IONPs functionalization

IONPs were coated with citrate using the protocol described by Campelj et at.[2,3] These were coated with citric acid to create a negative surface charge to prevent particle aggregation. PAC-1 antibody was dialysed prior to conjugation to remove sodium azide using a Pur-A-Lyzer™ Mini Dialysis Kit (Sigma) following the guidelines of the manufacturer (BD Bioscience). The PAC-1 antibody was then covalently attached to the citrated surface by carbodiimide activation of the carboxyl group of citric acid. 3.2 g_{Fe} mL^{-1} of citrated IONPs were activated using 0.96 mg mL^{-1} EDAC and 1.92 mg mL^{-1} NHS dispersed 0.1 MES buffer (Sigma) at pH 6.3 for 1 hour at RT on an orbital shaker. Activated IONPs were then washed 3 times using 0.1 MES buffer at pH 6.3, re-suspended in 1x PBS containing 20 μg mL^{-1} PAC-1 antibody and left overnight at 4°C in constant orbital shaking. The next day, 200 μM glycine dissolved in PBS was added to the dispersion and incubated for 45 minutes at 4°C. Finally, IONPs were washed 5 times with 1 x PBS by centrifugation. A similar methodology was followed to fabricate fluorescent f-IONPs (fluo-f-IONPs) but using a fluorescent version of the PAC-1 antibody (antibodies.com). Successful conjugation of the PAC-1 antibody to the citrate-coated IONPs was confirmed by different complementary means as shown here as well as in the main manuscript. Dynamic light

scattering and electrophoretic light scattering, measured with a ZetaSizer-Nano (Malvern, UK) in water at 0.05 g_{Fe} L^{-1}, showed that after conjugation the functionalised IONPs (f-IONPs) had an increased hydrodynamic size (154 to 180 nm; PDI 0.331 to 0.378, Fig. S1) and a reduction in surface negative change (-39.1 ± 0.3 to -37.2 ± 0.4 mV). To assess the conjugation visually, 0.55 g_{Fe} L^{-1} of either IONPs or f-IONPs dispersed in 100μL 1x PBS was incubated with 6 μg of fluorescently labelled anti-IgM antibody (Goat Anti-Mouse IgM mu chain, Alexa Fluor® 488, Abcam PLC, UK) for 1 h at RT under orbital stirring. Later, the particles were washed two times with 1x PBS and observed using an Olympus FluoView FV 1200 microscope using an excitation wavelength of 473 nm, and emission wavelengths of 490-520 nm (Figure 1C). Additionally, fluorescent measurements at 528 nm were obtained from these same samples using a plate reader (Figure 1D). UV-Vis spectroscopy was measured in 0.1 M PB solutions containing 0.1 g_{Fe} L^{-1} of either IONPs or f-IONPs using a Agilent Cary 60 UV spectrophotometer (Figure 1E). All the results (Figure 1) are consistent with covalent bonding of the PAC-1 antibody to the activated –COOH groups on the surface of the IONPs.

f-IONPs labelling of activated platelets and ex vivo generated blood clots

A microplate-based assay was used to assess the ability of f-IONPs to bind to activated platelets. A 96 well-plate was coated with poly-L-lysine for 1 hour at RT and then washed three times with HEPES-buffered saline (HBS; pH 7.4, 145 mM NaCl, 10 mM HEPES, 5 mM KCl, and 1 mM $MgSO_4$). Wells were then incubated for 10 minutes at RT with washed human platelet suspensions ($2x10^8$ cells mL^{-1}) resuspended in supplemented HBS (HBS including 10 mM D-Glucose and 1mg mL^{-1} BSA). The samples were then washed and exposed to either 100 uL of supplemented HBS alone, or supplemented HBS containing 0.1 U mL^{-1} thrombin for 2 minutes at 37°C. Samples were then fixed by addition of 1% [v v^{-1}] formaldehyde, and stored at 4°C until use. The supernatant was removed, and the samples were blocked for 1 h at RT with Normal goat serum (Abcam, UK). This was removed and the samples were incubated for

1 h at RT in either phosphate buffered saline (PBS) alone, or PBS containing 0.05 g_{Fe} L^{-1} f-IONPs. The wells were then washed 3 times with PBS on an orbital shaker for 5 mins at RT and incubated with a 1:500 dilution of goat anti-mouse Alexa Fluor® 488-labelled anti-IgM mu chain antibody (Abcam, UK) in PBS at RT for 1h. The samples were then washed 3 times further with PBS, and then measured fluorometrically with a BioTek 2 synergy microplate reader using excitation wavelengths of 475-495 nm, and emission wavelengths of 518-538 nm.

Alternating Current (AC) susceptometry: 0.33 g_{Fe} L^{-1} f-IONPs was incubated at 37 °C for 10 min in either 200 µL of a PPP sample alone, or a PPP sample containing an *ex vivo*-derived human blood clot made from the same donor. Subsequently, AC susceptibility of the samples was recorded in a custom-made AC susceptometer system to determine the immobilization of the magnetic nanoparticles on the clot surface, as have been previously described for other cells (Figure 2B) [4-6].

Confocal imaging: Blood clots were then incubated with 0.33 g_{Fe} L^{-1} fluo-f-IONPs for 10 minutes at 37 ° C, washed three times with phosphate-buffered saline (PBS) and observed under confocal microscopy to assess a fluorescent version of PAC-1 labelled IONPs, binding to the clot surface (Figure 2C). This was observed using an Olympus FluoView FV 1200 microscope using an excitation wavelength of 473 nm, and emission wavelengths of 490-520 nm.

f-IONPs magnetic and thermal response under AMF

AC hysteresis loops were measured using an AC Hyster magnetometer (Natonech Solutions S.L., Spain) in 300 µL water solutions containing 2 g_{Fe} L^{-1} of either non-functionalized (IONPs) or PAC-1 functionalized (f-IONPs) nanoparticles under 100 kHz and 30 mT AMF (Figure 2D). Specific absorption rate (SAR) was measured using a NanoHeat field generator (Nanoscience Laboratories Ltd, UK) and an optical temperature probe, according to the method described elsewhere[7]. Using 200 µL water solutions containing 2 g_{Fe} L^{-1} of f-IONPs, SAR was measured at four different magnetic field conditions (105 kHz – 46 mT, 156 kHz – 42 mT; 205 kHz – 40

mT and 306 kHz – 30 mT; Figure 2F). Temperature curves of 200 µL PPP alone or containing 0.33 g_{Fe} L^{-1}, 0.089 g_{Fe} L^{-1} or 0.021 g_{Fe} L^{-1} f-IONPs; exposed to an AC magnetic field of 306 kHz and 30 mT for 30 min, were recorded using the same later instrumental set-up (Figure 2G). To prevent excessive heat exchange from the AMF to the sample, the glass utilized as a sample holder was isolated with a polysterine shield (Figure 2E).

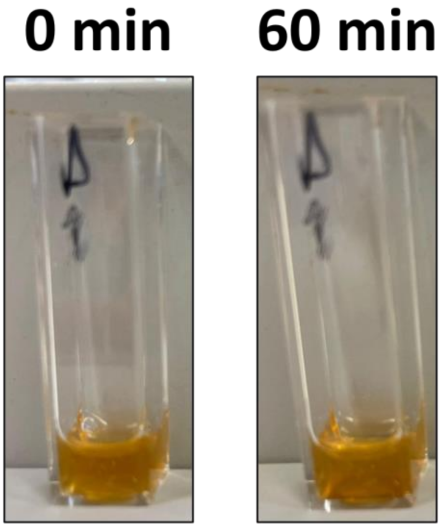

Figure S1. f-IONPs remain stable in PPP suspension for 60 minutes. 0.089g_{Fe}/L IONPs were diluted in PPP from three different donors and pictures were taken every 5 minutes. No f-IONPs agglomeration and precipitation was found. n = 3

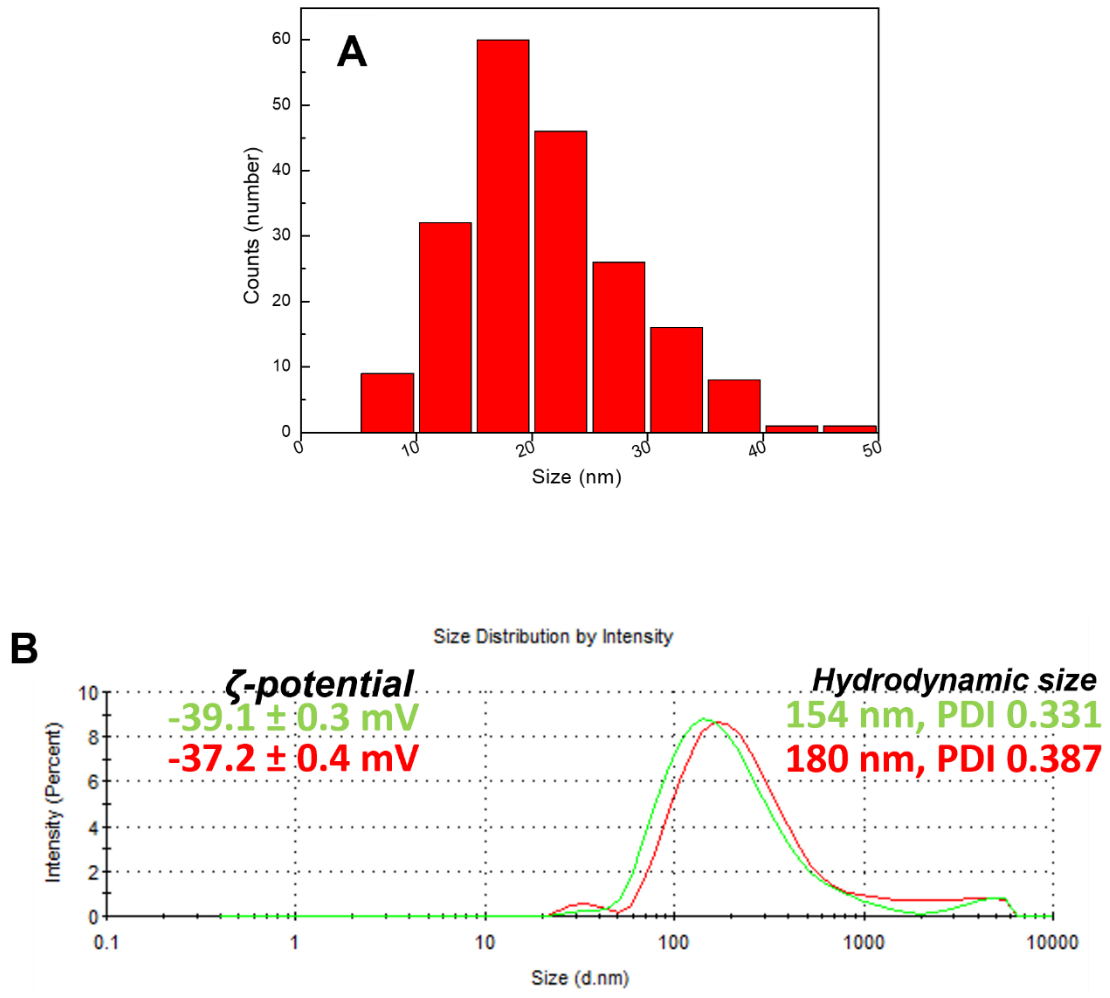

Figure S2. A) Size distribution of f-IONPs. B) Hydrodynamic size diagram and zeta potential values of f-IONPs before (green colour) and after (red colour) functionalisation PAC-1 antibody.

OTOTO fixation method for scanning electron microscopy

Fixed clots were washed with 0.1 M sodium cacodylate/2 mM $CaCl_2$ buffer three times, post-fixed with 1% OsO_4 in similar buffer for 1 h and washed six times with distilled water. Later, the clots were impregnated with osmium tetroxide by incubation in a saturated aqueous solution of sodium thiocarbohydrazide for 20 minutes, intermediate washing with distilled water six times and incubation again with 1% OsO_4 for two hours. Clots were then washed six times with distilled water and the impregnation process were repeated again. Later, the clots were dehydrated through a graded ethanol series of increasing concentration (70%, 80%, 90%, 100% and dry 100%) for 30 minutes in each concentration. Dry ethanol was then removed from the

clots using a critical point drier and adhered to scanning electron microscopy (SEM) supports with adhesive carbon planchets (Agar Scientific).

Effects of increased macroscopic temperatures in thrombolytic activity of tPa in ex vivo human blood clots

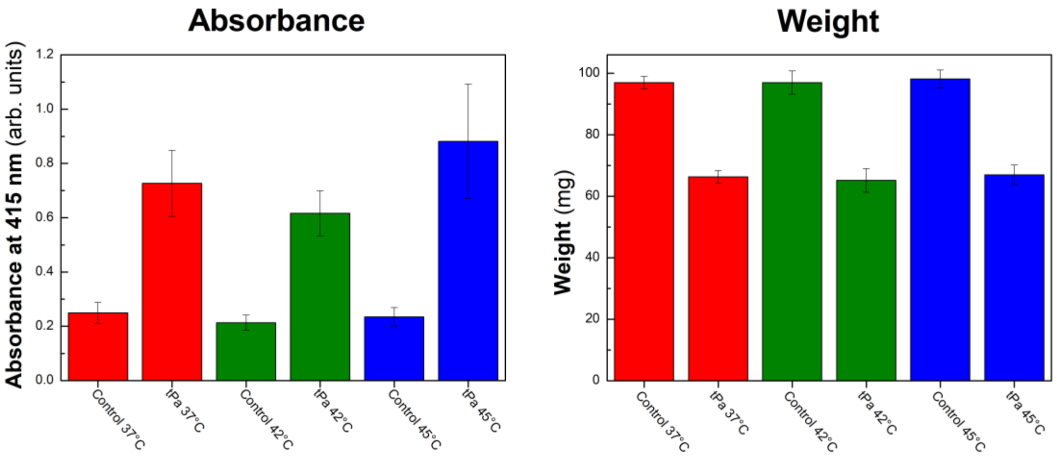

Figure S3. Absorbance measurements (415 nm) of the supernatant of the sample after the and mean clot weight of clots untreated or treatead with tPa, incubated at 37, 42 and 45°C for 30 min in absence of f-IONP and MH. No significant differences ($P > 0.05$) were found between tPa treatments at different incubation temperatures. n = 6, error bars indicate SErM.

Effects of increased macroscopic temperatures in PRP clots permeability to fluorescently-labelled dextran

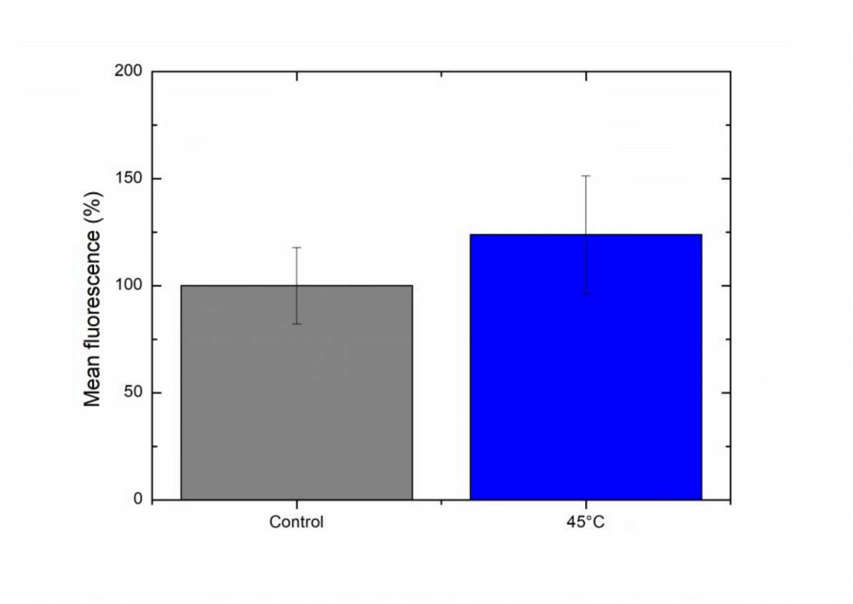

Figure S4. Raised macroscopic temperature does not significantly increase permeability of *ex vivo* derived PRP thrombi. Thrombi were fabricated by ADP-stimulation of platelet-rich plasma (PRP). Thrombi were incubated at 37°C (control) or 45°C for 30 minutes, in the absence of both f-IONPs and an AMF. The thrombi were then exposed to an HBS solution containing 3 µM Rhodamine-B-labelled 70 kDa dextran. After 2 minutes of incubation at RT, the penetration of dextran into PRP clots was assessed by performing z-scans using an Olympus FluoView FV 1200 confocal microscope using a slice depth of 20 µm, excitation wavelength of 473 nm and 543 nm, and emission wavelengths of 490-520 and 590-620 nm. 3D images were reconstructed, and mean slice dextran fluorescence was measured using ImageJ software. No significant differences in permeability were found between both groups ($P > 0.05$). n = 6

Effect of IONPs and IONPs-mediated MH in coagulation time

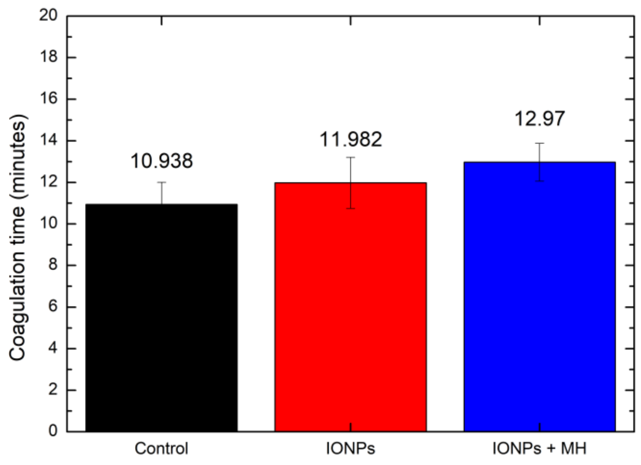

Figure S5. Magnetic hyperthermia does not significantly modify coagulation time in samples exposed to IONPs and IONPs and magnetic hyperthermia. Citrated whole blood was recalcified (20 mM $CaCl_2$) and incubated either alone or with $0.089 g_{Fe}/L$ IONPs at 37° C for 30 minutes while a third set of samples was exposed to $0.089 g_{Fe}/L$ IONPs-mediated MH for a similar time. Coagulation was assessed by visual observation. ($P > 0.05$). n = 5

1. Carpenter CE, Ward RE. Iron Determination by Ferrozine Method. In: Nielsen SS, ed. Food Analysis Laboratory Manual. Cham: Springer International Publishing; 2017:157-159.
2. Campelj S, Makovec D, Drofenik M. Preparation and properties of water-based magnetic fluids. *Journal of Physics: Condensed Matter*. 2008;20(20):204101.
3. Răcuciu M, Creangă DE, Airinei A. Citric-acid-coated magnetite nanoparticles for biological applications. *The European Physical Journal E*. 2006;21(2):117-121.
4. Cabrera D, Coene A, Leliaert J, et al. Dynamical Magnetic Response of Iron Oxide Nanoparticles Inside Live Cells. *ACS Nano*. 2018;12(3):2741-2752.
5. Soukup D, Moise S, Céspedes E, Dobson J, Telling ND. In Situ Measurement of Magnetization Relaxation of Internalized Nanoparticles in Live Cells. *ACS Nano*. 2015;9(1):231-240.
6. Moise S, Céspedes E, Soukup D, Byrne JM, El Haj AJ, Telling ND. The cellular magnetic response and biocompatibility of biogenic zinc- and cobalt-doped magnetite nanoparticles. *Scientific Reports*. 2017;7:39922.
7. Teran FJ, Casado C, Mikuszeit N, et al. Accurate determination of the specific absorption rate in superparamagnetic nanoparticles under non-adiabatic conditions. *Applied Physics Letters*. 2012;101(6):062413.
